# Microdissection TESE versus conventional TESE for men with nonobstructive azoospermia undergoing sperm retrieval

**DOI:** 10.1590/S1677-5538.IBJU.2022.99.14

**Published:** 2022-03-14

**Authors:** Sandro C. Esteves

**Affiliations:** 1 ANDROFERT, Clínica de Andrologia e Reprodução Humana Campinas SP Brasil ANDROFERT, Clínica de Andrologia e Reprodução Humana, Campinas, SP, Brasil; 2 Universidade Estadual de Campinas Departamento de Cirurgia (Disciplina de Urologia) Campinas SP Brasil Departamento de Cirurgia (Disciplina de Urologia), Universidade Estadual de Campinas - UNICAMP, Campinas, SP, Brasil; 3 Aarhus University Faculty of Health Denmark Faculty of Health, Aarhus University, 8000 Aarhus C, Denmark

## INTRODUCTION

Sperm retrieval techniques are classically used to harvest sperm from the epididymis and testis of men with azoospermia seeking fertility (1). After a successful sperm retrieval, intracytoplasmic sperm injection (ICSI) is mandatory because epididymal and testicular sperm cannot fertilize oocytes by conventional in vitro fertilization (IVF) (2).

For men with nonobstructive azoospermia (NOA) undergoing sperm retrieval (SR), the testis is the target organ because sperm production, if present, is generally minimal and restricted to the seminiferous tubules (1, 3). Both percutaneous and open methods can be used to harvest sperm from the testis of these patients. These methods are widely known by their acronyms TESA (testicular sperm aspiration), TESE (testicular sperm extraction), and microdissection TESE (microdissection testicular sperm extraction), based on the use of percutaneous or open approaches and whether or not microsurgery is utilized (1).

TESA relies on percutaneous needle aspiration, usually using a large needle connected to a syringe. The needle is inserted into the testis, negative pressure is created, and the tip of the needle is moved within the testis to disrupt the seminiferous tubules and sample different areas (1, 4). Conventional TESE (cTESE) relies on single or multiple open testicular biopsies carried out without magnification. By contrast, microdissection TESE (mTESE) relies on an operating microscope and microsurgical technique to identify and extract seminiferous tubules more likely to contain sperm (1, 4).

Despite being a relatively simple procedure, needle aspiration should be the last option for men with NOA. The reasons relate to the twice lower sperm retrieval rates (SRR) reported for TESA (~10-23%) compared with cTESE and mTESE (~40-50%) (4-7). Moreover, complications are more frequent with TESA, and according to some studies, it may affect up to 24% of patients (5).

Even if TESA is successful, the number of harvested sperm is typically lower than cTESE and mTESE, limiting the availability of sperm for cryopreservation (8). For these reasons, we do not support the routine use of TESA for NOA males. However, TESA is still carried out, for example, in low resource centers, particularly in patients with a history of previous positive retrieval by TESA or a biopsy report showing hypospermatogenesis (7, 9).

## WHAT GUIDELINES RECOMMEND

According to the 2021 American Urological Association (AUA)/American Society of Reproductive Medicine (ASRM) Guidelines on ‘Diagnosis and Treatment of Infertility in Men’, mTESE should be performed in men with NOA undergoing sperm retrieval (10). By contrast, cTESE or mTESE is the technique of choice according to the 2021 European Association of Urology (EAU) Guidelines on ‘Male Sexual and Reproductive Health’ (11).

The evidence supporting the AUA/ASRM guideline relates to the findings of two systematic reviews directly comparing cTESE and mTESE (6, 12). Based on data compilation of seven studies providing a direct comparison between both techniques, including 1254 patients, the pooled SRR was 52% for mTESE and 35% for cTESE, meaning that mTESE was 1.5 times more likely to result in successful SR than cTESE (6). The SRR ranged from 42.9% to 63% in mTESE versus 16.7% to 45% in cTESE (12). The differences were statistically significant in five of seven studies. Interestingly, in a subanalysis by histopathology of specimens taken during the operations, mTESE performed better in all categories but more remarkably among men with Sertoli cell-only (SCO) (6, 12).

By contrast, the evidence supporting the EAU guideline relates to the results of a large meta-analysis by Corona and co-workers published in 2019 (13). In this study, the authors included over 100 studies using either mTESE alone, cTESE alone, or both procedures, accounting for over twenty thousand patients with presumed NOA. Corona et al. reported an overall SRR of 47%, with no differences between cTESE and mTESE techniques.

## A CRITICAL APPRAISAL OF EXISTING EVIDENCE

Although we should commend Corona and co-workers for their efforts in undertaking such an exhaustive review of the available data, their results indicating similar effectiveness using either method was based, overwhelmingly, on trials that did not directly compare both techniques. These trials, in most cases, reported SRRs one way or the other, using different patient populations (13). Therefore, their study's design was entirely different from meta-analyses compiling data of studies directly comparing cTESE and mTESE.

The methodological issue mentioned above was raised in a letter to the editor of Human Reproduction Update, which pointed out several other concerns in the study by Corona et al. (14). First, traditional meta-analytic techniques assume that effect sizes are independent. However, the reported effect sizes (i.e., SRR) are likely to be different for non-comparative trials because the studied populations vary and likely be heterogeneous. Since SRRs relate to the specific population from the primary studies, this structure creates dependence, and the pooled results based on conventional meta-analytic methods might be misleading. Indeed, we noticed substantial evidence of bias in the reporting of Corona et al., as up to 38% of mTESE trials involved selected patient populations with an unfavorable prognosis, such as patients with previous failed SR or men postchemotherapy (14). By contrast, only ~6% of the cTESE trials included the so-called unfavorable patients, thus possibly overestimating the SRR for cTESE.

Second, although it is well-known that SRR depends on histopathology results, with poorer outcomes for SCO patients than maturation arrest and hypospermatogenesis (15, 16), the study of Corona et al. was not controlled for this critical confounding factor. We carefully analyzed the studies included in the above meta-analysis and found that the proportion of patients with SCO or tubular atrophy was significantly higher in mTESE trials than cTESE trials (57.3 vs. 46.8%) (14). We, therefore, reassessed the SR estimates, pooling the data of only non-comparative studies that provided diagnostic histopathology details. On this basis, we found that, overall, mTESE resulted in significantly higher SRR than cTESE (50.3 vs. 47.4%, p=0.002) (14). Additionally, when the analysis was limited only to patients with SCO, mTESE resulted in a significantly higher SRR than cTESE (34.7% vs. 31.2%, p=0.019) (14).

We also compiled the data of controlled studies directly comparing mTESE versus cTESE. In this analysis, the differences were even higher in favor of mTESE. Overall, the relative risk (RR) of finding sperm was 1.35 times higher using mTESE (95% confidence interval [CI]: 1.14 to 1.61; p=0.0003) (14). Our analysis of controlled studies indicated that the number of patients needed to be treated (NNT) by mTESE (vs. cTESE) to obtain one additional positive SR was 7.6 (95% CI: 5.0-16.6) (14). Moreover, mTESE was even more advantageous in patients with the worst histopathology phenotype, i.e., SCO (SRR: mTESE 36.1% vs. cTESE 13.3%; RR: 2.70, 95% CI: 1.72 to 4.24; p<0.0001). In SCO patients, the NNT by mTESE (vs. cTESE) to obtain one additional successful SR was only 4.4 (95% CI: 3.2 to 7.1) (14).

Collectively, our reanalysis of Corona et al. data (13) showed that the SRR is indeed affected by the surgical technique, provided strict diagnostic criteria are applied to identify the NOA patient. Notably, the higher the study's quality (i.e., controlled trials), the higher the magnitude of the effect size, as fewer patients need to be treated by mTESE vs. cTESE to achieve one additional positive SR when data of controlled studies (vs. non-controlled studies) are compared (14). Since SR success depends on many different factors, including patient selection criteria, surgeon's experience, embryologist's expertise, and laboratory technique to process retrieved specimens, only studies directly comparing cTESE versus mTESE can be considered in a meta-analysis assessing the effectiveness of these techniques.

Although further studies would be certainly welcomed in this area, mainly randomized controlled trials, there exists level 1 evidence from well-performed meta-analyses that compiled data of studies directly comparing mTESE vs. cTESE (6, 12). Based on these studies, there seems to be little question that mTESE provides optimized SR results in expert hands. Therefore, urologists should be judicious in interpreting the existing data as our ultimate goal is to deliver the best care to infertile men with NOA seeking biological parenthood.

## BEYOND SPERM RETRIEVAL RATES

Besides SRRs, other endpoints to consider in studies comparing mTESE vs. cTESE include complication rates, quantity and quality of sperm collected, and ICSI outcomes. In a 2021 study, we summarized the published evidence on the most relevant endpoints using nearly 120 articles (17). We found that in the general population of NOA patients who have not undergone previous SR (naïve population), the pooled SRR by mTESE was 46.8%. Additionally, in studies reporting SR by mTESE for men with a history of failed TESA or cTESE, the SRR was 39.1%.

We also showed that mTESE was associated with an overall 2.6% complication rate (17). The reported complications were mainly minor and included persistent pain, infection, and hematoma. But a few cases of testicular fibrosis and atrophy were reported following mTESE. Importantly, in controlled studies directly comparing the techniques, the complication rate was lower using mTESE than cTESE (1.3% vs. 3.0%, respectively), attributed to less testicular tissue extraction and preservation of intra-testicular blood supply (17).

Consistent with the above findings, fewer complications have been reported on ultrasound examination after mTESE than cTESE (12). Also, the amount of tissue extracted has been reported to be lower in mTESE vs. cTESE (12, 18). But notably, in the cases where mTESE is aimed for sperm cryopreservation or when sperm-producing seminiferous tubules are minimal, it may be necessary to remove larger quantities of testicular parenchyma that may equal or even exceed that of cTESE. In these cases, the advantages of mTESE relate to a richer harvest or a poor but positive sperm recovery.

Concerning ICSI outcomes, our review mentioned above indicated that fertilization rates and pregnancy outcomes with testicular sperm retrieved by either mTESE or cTESE were inconsistently reported. Of note, no published data exist on these endpoints from studies directly comparing the techniques. Nevertheless, polled data of studies using mTESE alone indicate that the fertilization rate of testicular sperm by ICSI was about 57% (17). Along these lines, the pooled clinical pregnancy rate per embryo transfer cycle was 39%. Miscarriage, defined as the spontaneous loss of a clinical pregnancy before 22 completed weeks of gestational age, was seldom reported in the literature. Finally, live birth, defined as the delivery of at least one liveborn infant per transfer, was reported to be about 24%, but only a few articles provided data on live birth (17). These figures indicate that approximately one in four couples whose male partners had a successful sperm retrieval by mTESE take a baby home using ICSI with the patients’ sperm.

## OUR CLINICAL APPROACH

**Figure 1:**
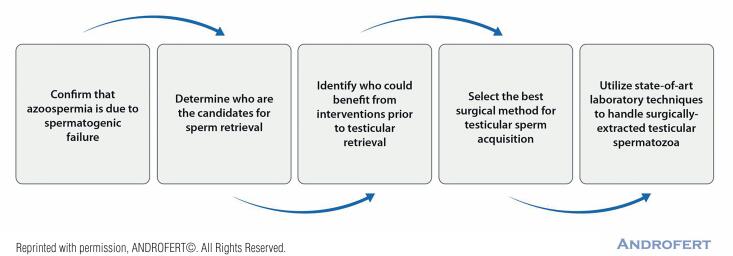
Step-by-step approach for the clinical management of men with nonobstructive azoospermia seeking fertility.

The clinical management of men with NOA seeking fertility has been a tremendous challenge for andrologists, urologists, and reproductive medicine specialists. We developed a five-step algorithm to most optimally manage these patients at our Clinic (Figure-1), detailed elsewhere (3). Briefly, it includes:

- Differential diagnosis with other types of azoospermia (19);
- Patient counseling about the chances of successful sperm retrieval and biological parenthood, which includes the use of genetic testing for diagnostic purposes and treatment guidance (20);
- Consideration for hormonal modulation or microsurgical varicocelectomy to increase sperm retrieval success in selected cases (9, 19, 21);
- Application of the most effective and efficient sperm retrieval technique (6, 12, 14, 22-24);
- Use state-of-art laboratory techniques for handling and freezing testicular sperm and cultivating the embryos resulting from testicular sperm injections (25, 26).

**Figure 2:**
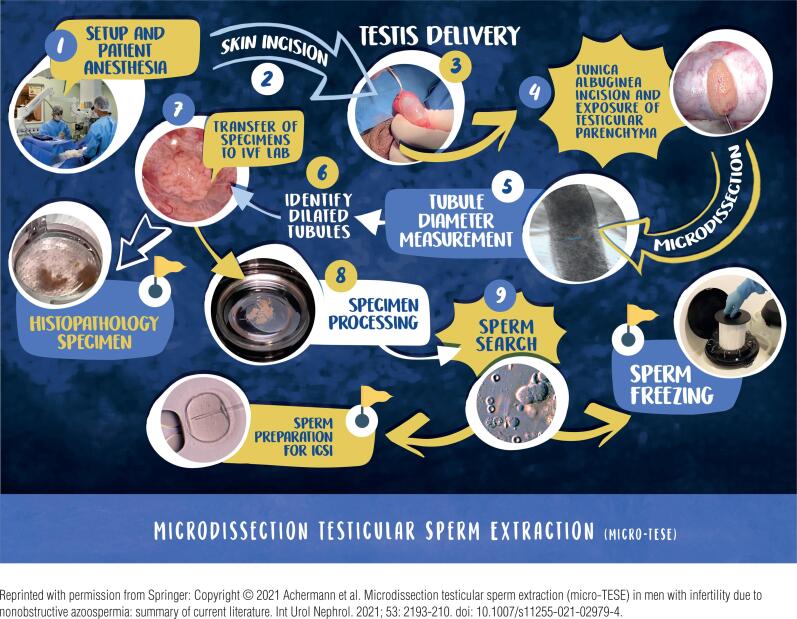
Microdissection Testicular Sperm Extraction Visual Map. The patient is brought shaved to the operating room, placed supine, prepped, and draped accordingly. Microdissection TESE is usually performed outpatient, under intravenously combined with local anesthesia (1). A transversal scrotal incision is fashioned (2), and the hemiscrotum is entered. The tunica vaginalis is opened, and the testis is delivered (3). An equatorial non-linear incision is fashioned in the tunica albuginea using a knife under operating microscopy at 6 to 8 times magnification (4). Microdissection is carried out through all areas of the superior and inferior poles of the testis. Magnification of 16 to 25-times is used when searching for the largest seminiferous tubules (5). Enlarged seminiferous tubules are identified (6 and 7), removed with micro-forceps, placed in a petri dish containing sperm culture medium (8), and sent to the IVF laboratory for examination (8). One or more specimens are taken for a histopathology examination. In general, the largest the tubule diameter, the greater the chance of finding active spermatogenesis (5). The extracted tubules are squeezed mechanically, and the cell suspension is examined under the inverted microscope in search of sperm (9). The surgeon is informed promptly if any sperm are found. Additional specimens can be taken to secure enough sperm for ICSI and freezing. The albuginea and vaginalis are closed, and the testicle is placed back to the hemiscrotum. Lastly, the dartos and skin layers are closed with absorbable sutures. The patient is discharged a few hours later.

In our hands, mTESE is the method of choice to harvest sperm from the seminiferous tubules of NOA males. A visual map of mTESE the way we do it at our Clinic is provided in Figure-2. A short movie illustrating the key operative and laboratory aspects of the procedure is available at <www.brazjurol.com.br/videos/may_june_2013/Esteves_440_441video.htm> (23).

Our facility includes two independent cleanroom IVF laboratories side-by-side, located next door to the operating theater (25). This setup allows embryologists to dedicate enough time for the NOA cases while the routine IVF/ICSI workload is taken care of in the other lab. We feel these details can make a difference. Indeed, our results with mTESE in a population of over one thousand men with NOA have been reassuring, with an overall SRR of 56%, varying according to the predominant testicular histopathology pattern (Hypospermatogenesis: 98%; Maturation arrest: 59%; SCO: 31%; Tubular sclerosis: 25%) (unpublished data). We believe that a state-of-art IVF lab, well-trained embryologists, good laboratory practices, and quality management are critical to optimizing embryonic and pregnancy outcomes. Overall, in a cohort of 912 ICSI cycles performed from 2007 to 2020 using testicular sperm retrieved from NOA males (average male age: 34.9 years; range: 23-64; average female age: 34.6 years; range: 21-44), two-pronuclei fertilization rates, blastulation rates, live birth rates, and cumulative delivery rates per aspirated cycle were 69.2%, 45.6%, 33.6%, and 44.2%, respectively (unpublished data).

**Figure 3:**
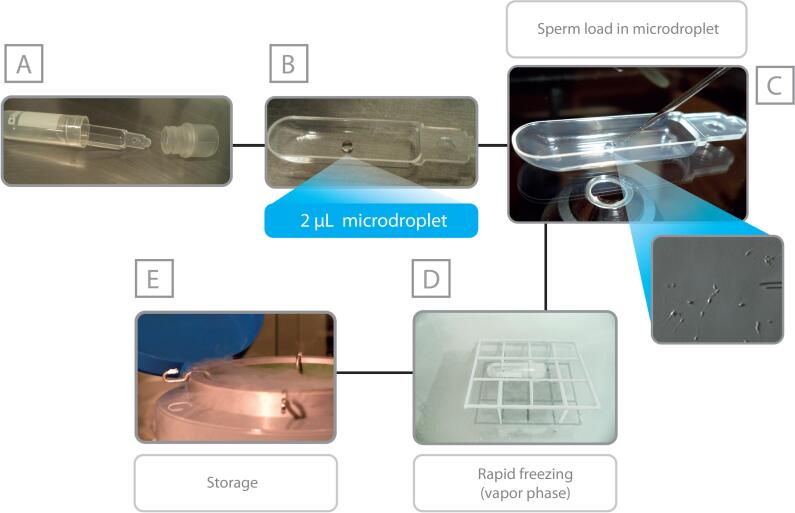
Testicular sperm cryopreservation using Cell-sleepers. The Cell Sleeper (Nipro, Japan) consists of an outer vial, an inner tray, and a screw cap (A). The inner tray is placed onto the lid of a large culture dish, and a 2-μ L droplet of cryopreservation solution is pipetted into the tray, in a central position (B). Spermatozoa are aspirated and ejected into the droplet with the aid of a microinjection pipette (C). Immediately after that, the tray is returned to the vial, and the vial is closed with the screw cap. The vial is placed in a horizontal position 4-5 cm above the surface of liquid nitrogen (D). After 2 min, the vial is submerged in liquid nitrogen and secured into a cryopreservation cane for long-term storage (E).

Currently, our preference is to perform mTESE as a separate procedure, before ovarian stimulation and oocyte pick-up. Sperm harvested from the seminiferous tubules are cryopreserved for future use. Our preferred method for freezing testicular sperm is vitrification using ‘The Cell Sleeper method’ (9, 27, 28). Briefly, Cell Sleepers consist of an outer vial, an inner tray, and a screw cap. Sperm are picked up with the microinjection pipette and ejected into the droplet on the tray. The tray is placed into the vial, and the vial is firstly frozen on liquid nitrogen vapor, then submerged in liquid nitrogen for storage (Figure-3). This procedure is advantageous from a quality management perspective. It allows ICSI to be carried out using frozen-thawed testicular sperm without programming mTESE concomitantly to the oocyte pick-up. Our experience using fresh and frozen-thawed testicular sperm for injections indicates no significant difference in outcomes, therefore consistent with what has been reported in the literature (29).

**Figure 4:**
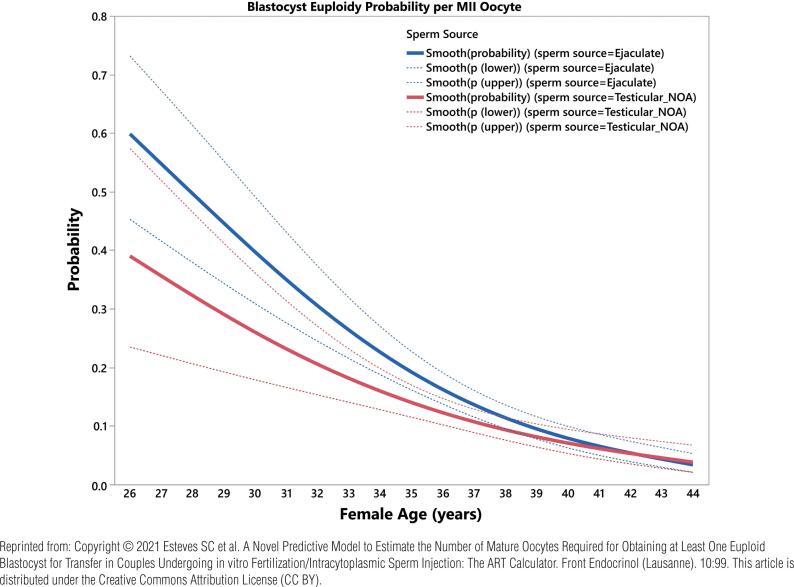
Blastocyst euploidy probability per metaphase II oocyte. The plots show the probability of a metaphase II (MII) oocyte turning into a euploid blastocyst as a function of female age. The estimated probabilities (solid curves) and their 95% confidence interval (dotted curves) are presented according to sperm source to be used for IVF/ICSI, namely, ejaculated sperm (blue) and testicular sperm extracted from patients with non-obstructive azoospermia (NOA) (red). The relations are non-linear and characterized by a differential modulatory effect of sperm source across age. The effect size of female age on blastocyst euploidy probability per MII oocyte from the year (t) to year (t+1) was defined as the ratio p(t+1)/p(t) × 100. There was a significant decrease (p<0.001) in the probability of an MII oocyte becoming a euploid blastocyst with aging.

**Figure 5:**
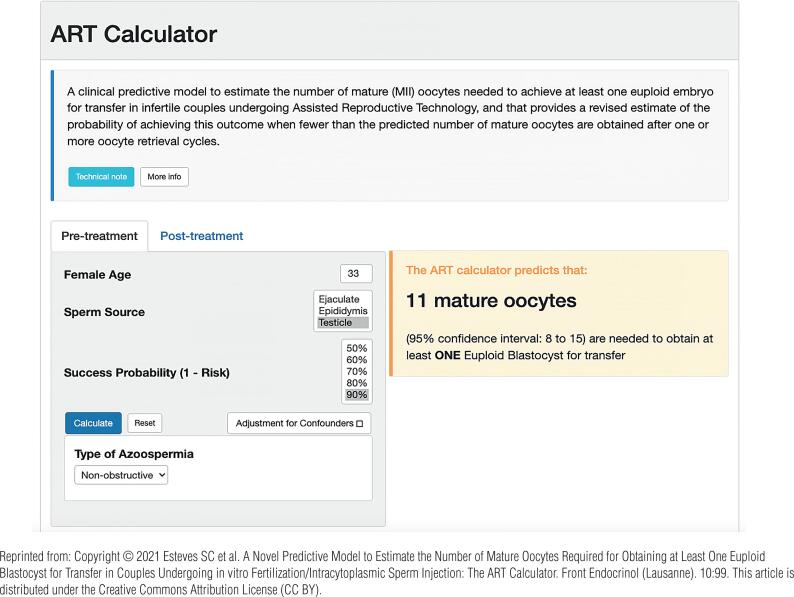
ART Calculator. Online calculator to estimate the minimum number of metaphase II oocytes required to obtain at least one euploid blastocyst for transfer in infertile patients undergoing IVF/ICSI cycles. The figure shows how the calculator is used in an office-based setting. (A) Pretreatment, clinicians input the patient's age and the sperm source for IVF/ICSI. If the option “Testicle” is marked, then the type of azoospermia (obstructive or nonobstructive) should also be defined. The user sets the probability of success for the estimation, which indicates the chance of having ≥1 euploid blastocyst when the predicted number of mature oocytes is achieved. Once the button “calculate” is pressed, a text box will pop up on the right side of the screen, indicating the predicted minimum number of metaphase II oocytes needed for obtaining at least one euploid blastocyst, with its 95% confidence interval. (B) Posttreatment, i.e., when fewer than the predicted number of metaphase II oocytes are obtained after one or more oocyte retrieval cycles. Clinicians input the pretreatment information and the actual number of metaphase II oocytes collected or accumulated. The user sets the probability of success; it reflects the chance of correct estimation according to the exact number of oocytes obtained. Once the button “calculate” is pressed, a text box will pop up on the right side of the screen, indicating the predicted probability of achieving ≥1 euploid blastocyst with the number of mature oocytes available. The ART calculator can be found online at http://www.members.groupposeidon.com/Calculator/.

Our research has show that the probability of having genetically normal blastocysts after ICSI is adversely affected by using testicular sperm taken from men with NOA (Figure-4) (30, 31). It means more oocytes are needed to obtain at least one euploid blastocyst for transfer in each couple undergoing ICSI with testicular sperm. Therefore, planning the ovarian stimulation regimen to increase oocyte yield is critical to improving the chances of biological parenthood for these couples. In our settings, we use a predictive model to estimate the number of metaphase II oocytes needed to obtain at least one euploid blastocyst for transfer in couples undergoing IVF-ICSI (Figure-5), which is particularly helpful for couples of NOA males (30).

## GAPS IN KNOWLEDGE

Although significant advances have been achieved in this area, there are still several knowledge gaps to be filled to improve our decision-making. We need more data from high-quality studies comparing cTESE and mTESE, controlling for relevant confounders, investigating SRRs and complications, quantity and quality of sperm collected, and ICSI outcomes, including the health of resulting offspring. We also need to know if there is a role for hormonal stimulation before SR and what type of patient might benefit from it. Further research is also warranted on predictors of SR success as it would be ideal for identifying who is eligible for SR, thus avoiding unnecessary operations. Lastly, we need to invest in better laboratory techniques to process and freeze testicular sperm and select the best sperm for injection. While we should certainly consider these limitations, they should not refrain us from using the best available evidence to guide our decisions in the best possible interest of our patients.

## CONCLUSIONS

Nonobstructive azoospermia represents the most challenging male infertility condition to manage. Despite that, it is not synonymous with sterility, as ~50% of the affected men have residual intratesticular sperm production. Sperm retrieved from the seminiferous tubules can be used for ICSI and result in viable offspring. An effective and safe SR technique is critical to offer these patients the highest chance of biological parenthood while preserving testicular function as much as possible. Microdissection TESE has been shown to fulfill these goals better than conventional TESE. Although SR is a critical element in the management of NOA males seeking fertility, the optimal management for the couple requires a coordinated multidisciplinary effort involving reproductive urologists, andrologists, reproductive endocrinologists, embryologists, and quality managers.
